# Addressing social needs in oncology care: another research-to-practice gap

**DOI:** 10.1093/jncics/pkae032

**Published:** 2024-04-27

**Authors:** Emily Haines, Rachel C Shelton, Kristie Foley, Rinad S Beidas, Emily V Dressler, Carol A Kittel, Krisda H Chaiyachati, Oluwadamilola M Fayanju, Sarah A Birken, Daniel Blumenthal, Katharine A Rendle

**Affiliations:** Department of Implementation Science, Wake Forest University School of Medicine, Winston-Salem, NC, USA; Mailman School of Public Health, Department of Sociomedical Sciences, Columbia University, New York, NY, USA; Department of Implementation Science, Wake Forest University School of Medicine, Winston-Salem, NC, USA; Department of Medical Social Sciences, Northwestern University Feinberg School of Medicine, Chicago, IL, USA; Department of Biostatistics and Data Science, Wake Forest University School of Medicine, Winston-Salem, NC, USA; Department of Biostatistics and Data Science, Wake Forest University School of Medicine, Winston-Salem, NC, USA; Perelman School of Medicine, Department of Medicine, University of Pennsylvania, Philadelphia, PA, USA; Perelman School of Medicine, Department of Surgery, University of Pennsylvania, Philadelphia, PA, USA; Penn Center for Cancer Care Innovation, Abramson Cancer Center, Philadelphia, PA, USA; Department of Implementation Science, Wake Forest University School of Medicine, Winston-Salem, NC, USA; Perelman School of Medicine, Department of Psychiatry, University of Pennsylvania, Philadelphia, PA, USA; Penn Center for Cancer Care Innovation, Abramson Cancer Center, Philadelphia, PA, USA; Perelman School of Medicine, Department of Family Medicine and Community Health, University of Pennsylvania, Philadelphia, PA, USA

## Abstract

Social determinants of health and unmet social needs are directly related to cancer outcomes, from diagnosis to survivorship. If identified, unmet social needs can be addressed in oncology care by changing care plans in collaboration with patients’ preferences and accounting for clinical practice guidelines (eg, reducing the frequency of appointments, switching treatment modalities) and connecting patients to resources within healthcare organizations (eg, social work support, patient navigation) and with community organizations (eg, food banks, housing assistance programs). Screening for social needs is the first step to identifying those who need additional support and is increasingly recognized as a necessary component of high-quality cancer care delivery. Despite evidence about the relationship between social needs and cancer outcomes and the abundance of screening tools, the implementation of social needs screening remains a challenge, and little is known regarding the adoption, reach, and sustainability of social needs screening in routine clinical practice. We present data on the adoption and implementation of social needs screening at two large academic cancer centers and discuss three challenges associated with implementing evidence-based social needs screening in clinical practice: (1) identifying an optimal approach for administering social needs screening in oncology care, (2) adequately addressing identified unmet needs with resources and support, and (3) coordinating social needs screening between oncology and primary care.

Social determinants of health and unmet social needs are directly related to cancer outcomes, from diagnosis to survivorship (1). Social determinants (ie, the “conditions in which people are born, grow, live, work, and age” (2)) affect the health and well-being of all. People with unmet social needs (eg, housing instability, food insecurity, exposure to violence, transportation difficulties) face inequities in cancer care. Failing to address the social needs of people with cancer can result in delayed diagnosis and treatment initiation, greater distress, higher acute care utilization, and increased risk of relapse and death (3-6). If identified, unmet social needs can be addressed in oncology care by changing care plans in collaboration with patients’ preferences and accounting for clinical practice guidelines (eg, reducing the frequency of appointments, switching treatment modalities) and connecting patients to resources within healthcare organizations (eg, social work support, patient navigation) and with community organizations (eg, food banks, housing assistance programs). The goal of addressing social needs in oncology encounters is to improve health outcomes for all cancer patients by mitigating health inequities.

Screening for social needs is the first step to identifying those who need additional support and is increasingly recognized as a necessary component of high-quality cancer care delivery (7,8). In the last 15 years, the number of publications focused on screening for social needs or social determinants of health has increased from 4 in 2007 to 106 in 2022 (a 26-fold increase) (9,10). Many screening tools have been developed and validated (11-13), as well as resources to improve the integration of screening into clinical practice. For example, the Social Interventions Research and Evaluation Network (SIREN) has compiled a library of more than 2000 publications, toolkits, and other resources on social needs screening intended for use in practice (9). Additionally, many electronic health records have standardized ways of storing identified social needs, including the Epic (©) Social Determinants of Health Wheel.

Despite evidence about the relationship between social needs and cancer outcomes and the abundance of screening tools, the implementation of social needs screening remains a challenge, and little is known regarding the adoption, reach, and sustainability of social needs screening in routine clinical practice (14-16). In this paper, we present data on the adoption and implementation of social needs screening at two large academic cancer centers and highlight three challenges associated with implementing evidence-based social needs screening in clinical practice. Such challenges must be addressed to close the research-to-practice gap and to facilitate health equity in oncology.

## Implementation of social needs screening in patients with cancer: what we learned

To examine the implementation of social needs screening, a cross-center initiative between two National Cancer Institute-funded P50 Implementation Science Centers in Cancer Control (ISC3)—Wake Forest University School of Medicine (WFUSM) and the Perelman School of Medicine at the University of Pennsylvania (Penn)—evaluated the uptake of an electronic health record module called the Epic (©) Social Determinants of Health Wheel (herein the “Wheel”). This study was approved by the Institutional Review Boards at WFUSM and Penn. The Wheel includes several domains for recording unmet social needs (eg, food insecurity, transportation needs, housing, financial strain). We assessed adoption of the Wheel among patients with cancer within the two healthcare systems and subsequently explored barriers and facilitators influencing Wheel implementation using key informant interviews with members of oncology care teams (clinicians and staff) as well as clinical leaders at one of the WFUSM sites (n = 6). *We found markedly low rates of routine adoption of social needs screening among patients with cancer (adoption defined as at least 1 of 6 social needs responses submitted for the Wheel)* (Table 1). Simultaneously, an inductive analysis of our key informant interviews revealed a commitment to measure and address social needs by oncology care teams. Based on our interview findings and extant social needs literature, we have identified several key challenges contributing to the gap between the perceived importance of measuring and addressing social needs among clinicians and leaders and limited adoption in practice.

**Table 1.** Epic (©). Social Determinants of Health (SDOH) Wheel completion rates (by domain) at Wake Forest University School of Medicine and University of Pennsylvania Health System among 87 855 patients diagnosed with cancer

Wheel domain	Domain completed (%)
Financial resource strain	6.0-9.6
Food insecurity	7.0-10.5
Housing instability	5.0^a^
Intimate partner violence	2.2-5.7
Social isolation	2.0-5.2
Transportation needs	5.8-47.6

All patients with a documented cancer diagnosis between January 1, 2019 and November 30, 2021 were first identified in the electronic health record (N = 87 855). We then queried data linked to the Epic (©) Wheel from two sources within the electronic health record: 1) internal social determinants of health patient registry; and 2) other structured data elements not associated with the registry but linked to the Wheel. The percentages represent the range of completion by domain across sources and sites.

a One institution did not provide data for this domain.

## Challenge 1: Is there an optimal tool or approach for administering social needs screening in oncology care?

Key informants at WFUSM described different approaches for integrating social needs screening into their settings that ranged from the implementation of standardized screening tools (eg, Epic (©). Wheel, National Comprehensive Cancer Network Distress Thermometer), to the creation of “home-grown” instruments, to more informal or ad hoc approaches (eg, tracking conversations about social needs in clinical notes). The implementation of a screening tool, whether standardized or developed for a specific site, can ensure that unmet social needs are more systematically identified across patients. Leveraging a standardized, validated screening tool can increase the reliability of screening and potentially offer more opportunity for comparisons across sites or across specialties within a healthcare system (ie, primary care vs oncology care). However, many such tools exist (17), making it difficult to determine which one to select, particularly in the absence of consensus about the core domains of social needs screening. With new requirements to report social determinants of health as a quality measure (18), measurement may become more standardized over time. For example, the Centers for Medicare and Medicaid Services now recommends five core domains: housing instability, food insecurity, transportation problems, utility help needs, and interpersonal safety (19).

Once a social needs screening tool has been selected, many decisions remain as to how best to administer it in routine care (20). Extant studies have disagreed on the optimal approach for routine delivery in terms of timing, workforce model, electronic vs paper format, and delivery in the clinic vs outside of the clinic, among other considerations (21,22). Furthermore, there is disagreement around the optimal frequency of reassessment of social needs as the need to account for evolving needs over the course of a patient’s care must be weighed against potential “survey fatigue” among patients who may be frustrated by the repetition of screening efforts.

In sum, there is a lack of consensus on the optimal approach to social needs screening administration, and the ideal approach may be setting-specific, making it difficult to offer generalizable guidance across settings. Given the wide variation in screening across settings, it may be more productive to identify the contextual factors (eg, staff bandwidth, existing workflows) that influence the administration of social needs screening most rather than identifying a single approach to administration. However, studying social needs screening administration across multiple settings may help to identify best practices. For example, a flexible approach to administration (eg, one in which patients are asked to complete screening tools at multiple time points and given options for completion through multiple platforms) may be preferred to account for patients’ varying preferences around screening administration (23).

## Challenge 2: How do we ensure that patients with identified unmet needs receive support and resources?

Merely assessing social needs is not enough to improve cancer outcomes or address some of the underlying factors that reinforce or exacerbate health inequities. Often, limited attention is given to the actionability of screening tools, including who is responsible for facilitating follow-up care (24-26). Failing to address identified social needs compromises patient outcomes and trust and decreases the acceptability of screening from the perspectives of patients and clinicians (27,28). It also raises questions around whether it is ethical to screen for social needs without taking any subsequent action to assist or intervene. To mitigate health inequities and improve outcomes, robust processes for following up on unmet social needs should be established. However, in some cases, even if the system cannot provide the “treatment” itself in addressing unmet social needs, awareness of patients’ social context can provide the opportunity to tailor care to the patient (29). For example, a provider might identify a medication that does not require refrigeration if the patient has unstable housing. Social needs screening also creates the opportunity for healthcare organizations to identify gaps in existing offerings to target with future service and resource development.

Addressing reported social needs first requires the systematic documentation of social needs in a location that is accessible to the clinicians and staff who are in a position to address them. This is a shortcoming in many current clinical environments. Second, it requires clinicians having the skillset and self-efficacy to use information regarding patients’ social needs in a clinically meaningful way. Third, it requires explicitly articulating setting-specific referral pathways or follow-up actions corresponding to each social need assessed. Creating a detailed catalog of existing services and resources available either in-house or through community partnerships can inform the identification of such referral pathways. Regarding resources available in the community, findhelp (formerly known as Aunt Bertha) (https://www.findhelp.org/) provides a national, web-based repository of food, health, housing, and employment resources which can be searched by ZIP code. There are also other resources such as 211 (https://211.org/) that can be tailored to resources available in a specific state. Although some studies have explored the potential automation of referrals (30,31), responding to reported social needs most often requires dedicated personnel (eg, social workers, navigators) who can triage needs, initiate referral pathways, and ensure that needs are met by services and resources to which a patient is referred in a patient-centered manner (22). This may be especially challenging in community oncology practices where specialty care may be more fragmented and where there may be fewer staff or resources to follow up on reported needs (32). Approaches for addressing social needs should account for setting-specific capacity and regional social service resources. Not all care settings need to screen *and* be the direct provider of social services; indeed, few settings will have the capacity to be end-to-end solutions. There is an urgent need to develop and test sustainable strategies that use a range of community and cross-section partnerships to ensure patients receive the support they need.

## Challenge 3: How do we better coordinate social needs screening between primary care and oncology?

At both ISC3 centers, we learned that most patients with cancer who did complete social needs screening did so as part of primary care rather than oncology care. Coordination of care between oncology and primary care is essential for high-quality care, and a known challenge due to myriad patient and system factors. We observed these same challenges in the context of social needs screening. For patients who enter the cancer center from external systems, oncology clinicians and staff may not be able to access social needs information collected during primary care appointments, leading to duplicative efforts and the potential to overburden patients with repetitive screening efforts. Even in the same healthcare system, the tools and pathways that primary care clinicians use to screen for social needs are not always the same as those used in oncology. For example, a primary care clinic may use the Wheel, while an oncology clinic in the same healthcare system may use the Distress Thermometer. Using different screening tools creates inefficiencies in screening and makes it difficult to assess patients’ responses over time. Unifying the tools within settings could optimize needs assessment and referral. A new trial at Penn seeks to identify the optimal intersection of delivery modality and instrument to facilitate social needs screening for patients with breast cancer, with the hopes of applying lessons learned to other areas of oncology and primary care (33).

Given that social needs are dynamic, particularly during cancer treatment when social needs may be exacerbated by the financial burden of treatment, relying solely on primary care assessment of social needs may create missed opportunities for identifying and addressing pressing social needs that could impact access to treatment and care outcomes. However, ignoring social needs assessment in primary care settings before or after cancer treatment may limit opportunities to reduce system and patient burden and understand social needs over time. As such, social needs screening should be studied at the system level to understand how to best harmonize efforts and share information across clinical and community settings. Importantly, patient perspectives on barriers to social needs screening should be incorporated into these efforts, a key limitation of our ISC3 study.

Taken together, our cross-center ISC3 study and other findings on social needs screening suggest a need for targeted multilevel efforts to enhance the widespread, routine, and equitable adoption, reach, and sustainability of efforts and tools to assess and address social needs in oncology. There may be advantages to standardizing the social needs information collected across settings; however, our findings suggest that endorsing a one-size-fits-all approach to administering and following up on social needs may not be appropriate. The broad contextual variation across cancer care settings (eg, varied organizational infrastructure and resources), as well as the variation in current screening processes (eg, screening tool-specific features, screening frequency), calls for a tailored approach to delivering implementation guidance. In practice, this means that strategies suggested to enhance the implementation of social needs screening should build on existing infrastructure and processes for social needs screening and follow-up and should be directly responsive to other key features of context (eg, staffing availability, workflow, resources, patient trust in sharing social needs with care teams). For example, we learned in our ISC3 study key informant interviews that social workers or electronic assessment of social needs may be infeasible in some settings but ideal in others.

Implementation science offers methods for generating such context-specific solutions to actively enhance implementation efforts and bridge the gap between research and practice (eg, Context-Driven Co-Design [CD2] (34), implementation mapping (35)). However, these methods require considerable time and resources to deploy on a setting-by-setting basis. As such, the relative advantage of such tailored implementation efforts compared to the provision of more generalized implementation guidance (or no guidance at all) should be empirically tested in relation to both implementation and patient outcomes. In the meantime, patient-centered efforts are needed to articulate the core functions of social needs screening and subsequent processes to address reported needs in oncology settings.
